# Transient Potential Profiling for Rapid Calcium Ion Quantification: Eliminating Conditioning Time in Solid-Contact Ion-Selective Electrodes

**DOI:** 10.3390/bios16060335

**Published:** 2026-06-12

**Authors:** Kaijie Zheng, Chenjie Yan, Mengwei Jiang, Jing Lei, Chengcheng Wang, Kai Zhao, Dajing Chen, Min Guo

**Affiliations:** 1 Shenzhen Angel Drinking Water Industrial Group Corporation, Shenzhen 518029, China; 2024112025088@stu.hznu.edu.cn (K.Z.); jing.lei@angelgroup.com (J.L.); chengcheng.wang@angelgroup.com (C.W.); kai.zhao@angelgroup.com (K.Z.); 2 School of Pharmacy, Hangzhou Normal University, Hangzhou 311121, China; 2025112025013@stu.hznu.edu.cn (C.Y.); 2024112025063@stu.hznu.edu.cn (M.J.)

**Keywords:** ion-selective electrodes, single exponential saturation model, reverse polarization

## Abstract

Traditional solid-contact ion-selective electrodes (SC-ISEs) are severely constrained by a long-standing thermodynamic bottleneck, which requires hours of pre-conditioning and stabilization to establish a stable phase-boundary potential. To fundamentally bypass this limitation, we present a paradigm shift in electrochemical ion sensing that exploits dynamic kinetics rather than waiting for thermodynamic equilibrium. In this paper, we report a transient potential profiling method that eliminates the need for equilibration by analyzing the open-circuit voltage decay during the first 60 s of polarization. A discharge step on indicator electrode returns the membrane to a reproducible initial state, allowing for the extraction of a concentration correlated coefficient. Using a calcium ISE with an optimized membrane, the early-stage polarization dynamics were fitted to a single exponential saturation model, predicting the steady state response with an average error of 1.6%. The method achieved high repeatability (intra-day RSD 3.22%), batch to batch reproducibility (4.57%), and recovery rates from 90.7% to 115.0% in real water samples. Validation against ion chromatography showed high agreement (R^2^ = 0.997). This strategy enabled conditioning free, disposable ISEs for point of care and environmental monitoring.

## 1. Introduction

The concentrations of alkali and alkaline earth metal cations, particularly sodium, potassium, and calcium, are critical indicators of water quality, directly governing electrolyte balance, hardness, and electrical conductivity [1,2,3,4]. Reliable quantification of these ions is indispensable across diverse applications, including food processing, sports physiology, aquaculture, environmental monitoring, and water treatment [5,6,7]. Among these, calcium (Ca^2+^) is of paramount significance, serving as the primary determinant of water hardness, which directly dictates industrial scaling risks and ecological health [8]. With the development of on-site rapid detection technology, higher requirements have been placed on the timeliness, accuracy, and portability of ion sensing platforms [9,10]. Especially in scenarios such as emergency response and process feedback regulation, the capacity to acquire rapid, real-time ion concentration data within a minimal operational window has become a frontier in sensor development [11,12,13,14].

Among available ion-detection methods, electrochemical approaches are the most widely used due to their simplicity, rapid response, and low cost [15,16,17]. In recent years, solid-contact ion-selective electrodes (SC-ISEs) have emerged as highly attractive candidates for portable systems due to their maintenance-free operation and ease of integration. Typically, these sensors utilize a polymeric ion-selective membrane (ISM) as the core sensing element to achieve quantitative analysis of target ions by covering its surface with a sensitive membrane that exhibits a selective response to specific ions [18,19]. Upon contact with the sample solution, a phase-boundary potential develops at the membrane–solution interface, which exhibits a linear dependence on the logarithm of ion activity, as described by the Nernst equation [18,20,21]. However, the practical deployability of SC-ISEs is fundamentally bottlenecked by the traditional conditioning protocol. Freshly prepared or dry membranes typically require prolonged soaking in a primary ion solution, often spanning several hours to days, to establish a stable phase-boundary potential [8]. Furthermore, upon sample introduction, practical measurements are severely hampered by polarization effects and slow interfacial charge-transfer kinetics [22,23,24]. This interfacial imbalance induces transient potential drifts that depart substantially from the theoretical Nernstian equilibrium [25]. Consequently, operators must endure tedious pre-conditioning cycles and wait tens of minutes for the potential to stabilize before recording reliable data, rendering SC-ISEs impractical for true plug-and-play or rapid on-site screenings [26,27].

To overcome these limitations, conventional research has focused on chemical modifications, membrane engineering, or mathematical compensation to accelerate equilibration or mitigate drift. For instance, using polymers with low swelling rates as membrane skeleton materials can reduce the hydration-induced interfacial instability [28,29], while adding surfactants to regulate the ion exchange behavior at the membrane–solution interface has slightly improved response consistency [30,31]. More direct attempts to bypass the conditioning phase include pre-loading the membrane cocktail with primary ions during fabrication to facilitate stoichiometric ion exchange without pre-soaking [32]. On the algorithmic front, advanced numerical models and neural network decoupling algorithms have been developed to correct for signal drift and multi-ion interference [33]. However, chemical additives often introduce risks of active ingredient leaching, compromising long-term selectivity [32], whereas algorithmic corrections require computationally intensive calibration protocols [33]. Ultimately, these approaches still focus on modifying equilibrium states or predicting stabilized values, failing to fundamentally bypass the time-consuming step of waiting for physical polarization to conclude [24,26,34].

**Figure 1:**
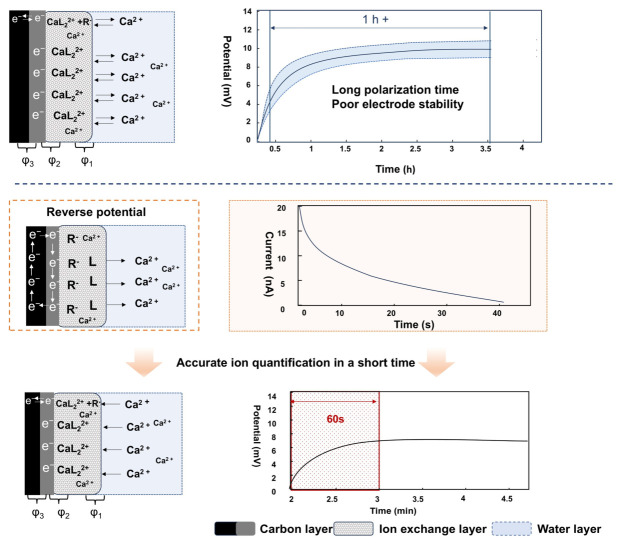
Working principle of reverse-polarization and ion concentration prediction.

Herein, we report a rapid ion detection method that leverages the dynamic electrochemical response during early-stage polarization rather than trying to eliminate it. Instead of waiting for the system to reach thermodynamic equilibrium, we systematically analyze the open-circuit potential as a function of time during the initial contact phase to extract a concentration-sensitive dynamic parameter—the transient relaxation constant. By establishing a robust functional relationship between this transient relaxation constant and the target ion activity, rapid concentration prediction is achieved without requiring full membrane polarization. This approach was successfully validated on a Ca^2+^-selective electrode optimized with tailored membrane materials and a reverse-polarization excitation protocol, enabling precise calcium quantification within 60 s (Figure 1). This strategy effectively bypasses the conventional conditioning requirement, providing a powerful, calibration-efficient tool for rapid, on-site water quality and biological fluid monitoring.

## 2. Materials and Methods

### 2.1. Chemicals and Materials

Calcium ionophore I (ETH 1001), potassium tetrakis(4-chlorophenyl)borate (KTClPB), 2-nitrophenyl octyl ether (o-NPOE), polypropylene (PP), and polyurethane (PU, Tecoflex SG-80A) were purchased from Sigma-Aldrich (St. Louis, MO, USA). Poly(vinyl chloride) (PVC) and tetrahydrofuran (THF) were obtained from Merck (Darmstadt, Germany). Calcium chloride dihydrate (CaCl_2_·2H_2_O), sodium chloride (NaCl), potassium chloride (KCl), and magnesium chloride hexahydrate (MgCl_2_·6H_2_O) were purchased from Sinopharm Chemical Reagent Co, Ltd. (Shanghai, China). All aqueous solutions were prepared with deionized (DI) water with a resistivity of 18.2 MΩ·cm. Screen-printed carbon electrodes (SPCEs) were prepared in-house by screen-printing carbon ink on PET substrates. Electrochemical measurements were performed using a CHI 1400A multichannel potentiostat (Shanghai Chenhua Instrument Co., Ltd., Shanghai, China), controlled by CHI electrochemical workstation software (version 12.04 or later, Shanghai Chenhua Instrument Co., Ltd., Shanghai, China).

### 2.2. Preparation of the Ion-Selective Membrane Cocktail

The ion-selective membrane (ISM) cocktail was prepared by dissolving the membrane components in 1 mL of anhydrous THF with a total mass of 100 mg. The optimized membrane composition (wt %) was as follows: 1.5 wt % calcium ionophore IV (ETH 1001), 1.0 wt % KTClPB, 32.2 wt % o-NPOE, and 65.3 wt % polymeric matrix (PP:PU = 2:1, *w*/*w*). The mixture was vortexed for 15 min and sonicated for 10 min at room temperature to ensure complete dissolution and homogeneous dispersion of all components. Prior to membrane casting, the cocktail was degassed under vacuum for 5 min to eliminate trapped air bubbles.

### 2.3. Fabrication of Solid-Contact Calcium Ion-Selective Electrodes

The SPCEs were first cleaned by rinsing with ethanol and DI water sequentially, followed by drying under a nitrogen stream. Specifically, 2 μL of the membrane cocktail was dispensed onto the center of the SPCE. The solvent was allowed to evaporate gradually under a gentle nitrogen flow at ambient temperature for 10 min, followed by vacuum drying at 60 °C for 1 h to ensure complete removal of residual THF and to stabilize the membrane matrix. The fabricated electrodes were stored in a desiccator under vacuum until use.

### 2.4. Electrochemical Preconditioning and Measurement Protocol

Prior to potentiometry, Ca^2+^-ISEs were preconditioned in 0.1 mM CaCl_2_ by applying a negative potential (−0.1 V to −0.6 V) vs. Ag/AgCl electrode for 10–40 s. Immediately after, open-circuit potential (OCP) was recorded using a CHI660 electrochemical workstation. Potential measurements were operated at room temperature with a screen printed Ag/AgCl reference electrode. In real sample analysis, Ca^2+^-ISEs were validated on commercial mineral water, diluted simulated serum (1:10 in Tris-HCl, pH 7.4), and tap water samples. Samples were analyzed without pretreatment. Results were compared with ICP-MS. Recovery tests with spiked Ca^2+^ assessed precision (RSD).

### 2.5. Preparation of Simulated Serum

Simulated serum was prepared by dissolving 8.035 g of NaCl, 0.355 g of NaHCO_3_, 0.225 g of KCl, 0.231 g of Na_2_HPO_4_, 0.311 g of MgCl_2_, 0.292 g of CaCl_2_, 0.072 g of Na_2_SO_4_, and 6.118 g of Tris-HCl in 1 L of deionized water, following a previously reported protocol with slight modifications [35]. Bovine serum albumin (BSA, Sigma-Aldrich, A1933) was added to the base formulation at a final concentration of 0.1% (*w*/*v*). The pH was adjusted to 7.4 if necessary. The simulated serum was freshly prepared, sterile-filtered (0.22 µm) where required, and stored at 4 °C prior to use.

## 3. Results

### 3.1. Surface Morphology and Characterization

**Figure 2:**
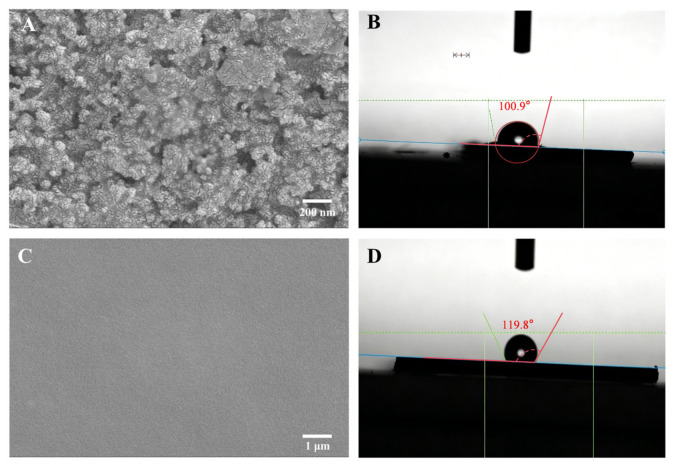
SEM and contact angle of screen-printed carbon electrode (**A**,**B**) and coated membrane (**C**,**D**).

The interfacial architecture between the solid-contact electrode and the ion-selective membrane plays a decisive role in governing both ion-to-electron transduction efficiency and long-term potentiometric stability. To elucidate the structural basis of the observed transient response behavior, scanning electron microscopy (SEM) and contact angle goniometry were employed to characterize the bare screen-printed carbon electrode and the membrane-coated electrode surface, as shown in Figure 2.

The carbon layer exhibited a distinctly rough and porous morphology, comprising interconnected carbon microparticles with irregular geometries and interstitial voids ranging from sub-micrometer to several micrometers in scale. This inherent surface roughness is advantageous for solid-contact ISEs, as it provides enhanced mechanical anchoring sites for the polymeric membrane and increases the effective electrochemical surface area available for charge transfer across the carbon–membrane interface. Correspondingly, the water contact angle measured on the coated membrane surface was 119° (Figure 2D), indicating a hydrophobic character of the ISM. The moderate hydrophilicity in Figure 2B facilitated the intimate wetting of the carbon substrate by the membrane cocktail during drop-coating.

### 3.2. Reverse-Polarization Optimization

**Figure 3:**
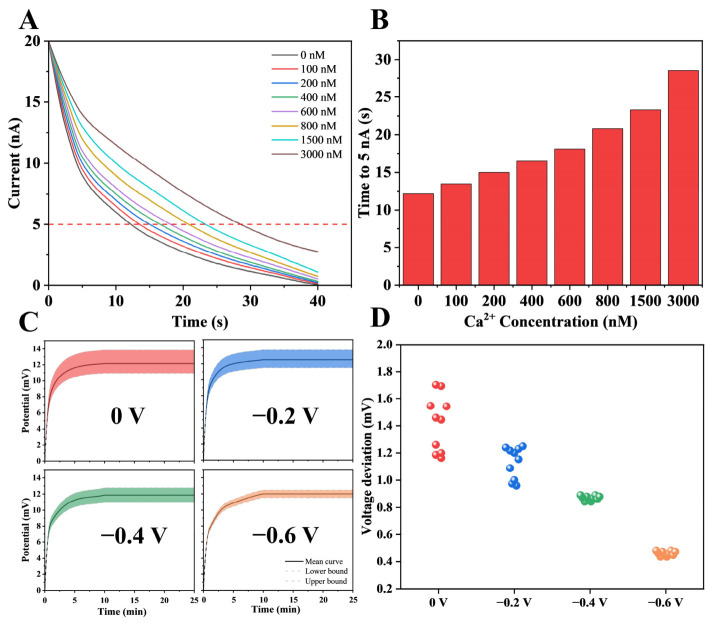
Optimization of reverse polarization protocols and its impact on transient response consistency. (**A**) Reverse polarization curves in varying Ca^2+^ concentrations. The dashed line at 5 nA marks the threshold current, enabling comparison of the time required to reach this value across different Ca2+ concentrations. (**B**) Comparison of reverse polarization duration to reach sub 5 nA current. (**C**) Open circuit potential transients following reverse polarization at different voltages. (**D**) Deviation analysis of open circuit potential transients.

The reliability of the transient-potential profiling strategy hinges fundamentally on establishing a well-defined initial state of the ion-selective membrane prior to each measurement. In this research, a reverse-polarization step on indicator electrode was introduced to expel residual primary ions from the membrane phase, thereby eliminating variability arising from uncontrolled ion distribution. Figure 3A showed the real-time current transient recorded during reverse polarization at −0.4 V in a conditioning solution containing different concentrations of Ca^2+^. The current observed in Figure 3A originates from transient ion-to-electron transduction during the reverse-polarization step. When an external potential is applied to the solid-contact electrode, the electrochemical equilibrium inside the membrane is perturbed, causing a redistribution of ionic species within the membrane, while compensating electronic charge redistribution simultaneously occurs in the conductive carbon layer. Upon application of the indicator electrode potential, the current exhibited a sharp initial surge exceeding 20 nA, attributable to the rapid electromigration of Ca^2+^ from the membrane bulk toward the electrode surface, followed by a monotonic decay as the membrane phase was progressively depleted of primary ions. Notably, the current stabilized below the 5 nA threshold after approximately 30 s, indicating that the majority of mobile Ca^2+^ carriers had been expelled from the membrane matrix and that the ion-exchange sites were predominantly occupied by the background electrolyte cations. This quasi-steady-state current likely reflected the residual background charging current and the limited leakage of Ca^2+^ from the outermost membrane boundary, rather than sustained bulk ion transport.

To examine the relationship between initial ion loading and depletion kinetics, the time required for the reverse-polarization current to decay below the 5 nA threshold was extracted from the transients and summarized in Figure 3B. Electrodes preconditioned in dilute CaCl_2_ solutions (0–600 nM) achieved the sub-5 nA baseline within 10–20 s, whereas those exposed to elevated concentrations (800–3000 nM) required longer than 20 s. This inverse correlation between pre-soaking concentration and depletion rate reflected the greater total inventory of Ca^2+^-ionophore complexes sequestered within the membrane matrix under high external loading. All curves converged to sub-5 nA beyond 30 s, confirming that a 30 s window is sufficient to make the membrane Ca^2+^-free.

The influence of reverse polarization voltage on the fidelity of subsequent open-circuit polarization transients was systematically evaluated following reverse polarization at 0.0, −0.2, −0.4, and −0.6 V (Figure 3C,D). At suboptimal voltages (0.0 and −0.2 V), incomplete expulsion of Ca^2+^ left residual ion–ionophore complexes within the membrane matrix, giving rise to poorly controlled internal ion activity. Consequently, the resulting polarization curves displayed broad error ranges and irregular decay morphologies, with mean absolute deviations of 1.5 mV and 1.1 mV, respectively. Such high variability, particularly during the critical 1–2 min window where curve fitting is performed, prevents reliable extraction of concentration-correlated dynamic parameters and renders these voltages unsuitable for rapid predictive quantification.

Quantitative deviation analysis (Figure 3D) confirmed these observations. The −0.4 V and −0.6 V conditions exhibited the low mean absolute deviation under 1 mV. At −0.6 V, reproducibility deteriorated further; excessive electrochemical stress exceeded the membrane stability window, potentially causing oxidative degradation of ETH 1001 or KTClPB and creating heterogeneous microdomains. These data established −0.4 V as the optimal pre-polarization potential, balancing exhaustive ion depletion with membrane integrity preservation.

### 3.3. Early-Stage Polarization Dynamics for Steady-State Prediction

To shorten the test equilibrium time, we investigated whether the transient open-circuit voltage recorded during the initial polarization phase could be used to predict the final steady-state response without awaiting full equilibration. To this end, voltage–time curves were recorded for five representative concentration levels, and three kinetic models were comparatively evaluated for their ability to extrapolate the equilibrium value from only the first minute of data.

**Figure 4:**
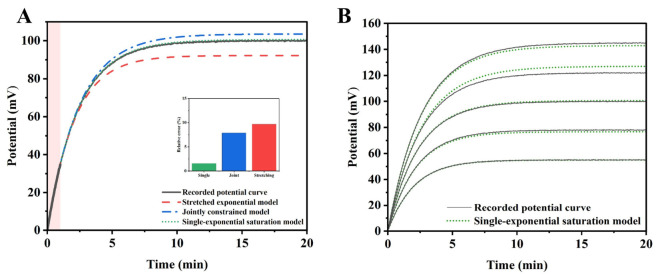
Early-stage polarization dynamics for steady-state prediction. (**A**) Comparison of three fitting models. The pink-shaded region denotes the data window used to constrain the fitting parameters and project the steady-state potential. (**B**) Single-exponential saturation model fitting in different Ca^2+^ concentrations.

As shown in Figure 4, the single-exponential saturation model, V(t) = A(1 − e^−kt^) (A > 0, k > 0) fitted independently to each curve, yielded the highest predictive accuracy. The estimated steady-state values (A) deviated from the experimentally recorded values by merely 0.11%, 1.74%, 0.64%, 4.09%, and 1.43% for the five concentrations, respectively, corresponding to an average relative error of 1.60%. Notably, the independently fitted time constants (τ = 1/k) were 6.02, 6.35, 7.05, 7.86, and 7.86 min, closely matching the underlying kinetic heterogeneity of each concentration level. This confirmed that once the early polarization trajectory captured approximately 10% of the full signal rise, the two parameters (A and k) were sufficiently constrained to permit robust extrapolation.

By contrast, enforcing a jointly constrained single-exponential model (Vi(t) = Ai(1 − e^−kt^)) across all curves degraded the average prediction error to 7.88%. The shared k value of 0.1372 min^−1^ (τ ≈ 7.29 min) represents a global compromise. It systematically overestimates the steady-state for fast-responding curves and underestimates it for slower ones. In the third mode, the stretched exponential model, V(t) = A(1 − e^−(t/τ)β^), generated an average error of 9.71%. These comparative results showed that the simple single-exponential saturation model, fitted independently per curve, provided the optimal balance between predictive accuracy and physical interpretability. The method effectively transformed the traditionally ‘wasted’ polarization period into an information-rich window. By recording the open circuit potential for only 1 min, the sensor’s steady-state response can be predicted with sub-2% average accuracy, thereby eliminating the need for prolonged pre-conditioning.

### 3.4. Interference and Water Layer Test

A water layer between the ion-selective membrane and the substrate promotes ion migration, causing potential drift and prolonged response, degrading electrode performance [25,36]. To test for such a layer, a water layer test was performed (Figure S1A). The electrode potential was recorded after immersing the electrode in 1 mM CaCl_2_ solution for 20 min, then in 1 mM NaCl solution for 20 min, and back to CaCl_2_ solution. The potential drifted upon switching solutions, but returned to near its initial value with a small offset, indicating no significant water layer at the interface.

The anti-interference capability of an ion-selective electrode is described by its selectivity coefficient $K_{i,j}^{\mathrm{pot}}$, which indicates the contribution of interfering ions to the potential. It was determined using the fixed interference method with the following formula:

$$K_{i,j}^{pot}=a_{A}/{\left(a_{B}\right)}^{z_{i}/z_{j}}$$

Here, $a_{B}$ is the activity of the interfering ion, and $a_{A}$ is the detection limit of the target ion at that activity. Figure S1B showed the selectivity coefficients of the electrode for Na^+^, K^+^, and Mg^2+^. All coefficients are below −3, indicating that the electrode has reliable anti-interference performance.

### 3.5. Repeatability and Stability Test

**Figure 5:**
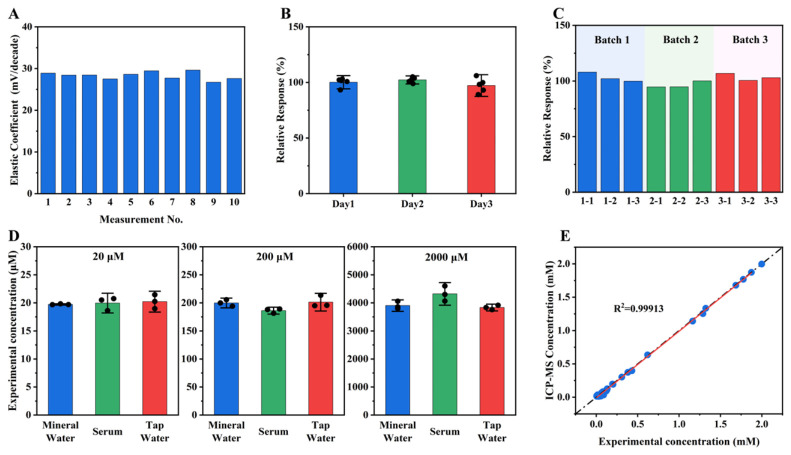
Electrochemical performance of fabricated sensor: (**A**) intra-day repeatability; (**B**) inter-day repeatability; (**C**) inter-batch reproducibility; (**D**,**E**) real water samples test.

The practical applicability of the proposed transient potential profiling strategy for rapid ion quantification depends critically upon its reproducibility across multi-measurements. As illustrated in Figure 5A, the intra-day repeatability was evaluated by recording the open circuit potential from ten consecutive measurements of 10^−4^ M Ca^2+^ using a single electrode. The obtained sensitivity exhibited a mean value of 28.30 mV/decade with a relative standard deviation (RSD) of 3.22%, which lies well within the acceptable range for potentiometric sensors and confirms that the transient-response profile remains highly consistent during continuous operation. This intra-day precision can be attributed to the effective electrochemical preconditioning step, which eliminates residual charge accumulation and establishes a reproducible phase-boundary potential prior to each transient recording.

Inter-day repeatability was further evaluated over a three-day period, during which the electrode was stored under vacuum in a desiccator between measurements and reconditioned prior to each daily measurement session. In Figure 5B, the relative responses remained stable with daily means of 100.19%, 102.27%, and 97.24% for Days 1, 2, and 3, respectively. The maximal drift between consecutive days was 4.92% (Day 2 to Day 3), satisfying the criterion of <5% per day. Notably, the slight downward trend observed on Day 3 may be ascribed to the gradual leaching of ionophore or plasticizer from the membrane matrix during prolonged storage, a phenomenon commonly reported for solvent polymeric membrane electrodes. Nevertheless, the overall stability over 72 h demonstrates that the proposed method maintained robust performance without requiring frequent recalibration, which is essential for on-site deployment. Inter-batch reproducibility was examined using three independent batches (three electrodes each; Figure 5C). Relative responses were 103.25% (RSD = 4.10%), 96.46% (RSD = 3.24%), and 103.40% (RSD = 3.11%), yielding an overall batch-to-batch RSD of 4.57%, well below the 10% threshold for disposable sensors. Drop-coating fabrication and controlled vacuum drying ensure uniform membrane thickness and component distribution.

### 3.6. Real Sample Validation

Spike-recovery experiments were performed using mineral water, simulated serum, and tap water samples (Figure 5D). Recovery rates ranged from 90.70% to 115.00%, satisfying the 85–115% criterion. Mineral water showed the smallest average S.D. (2.23%), reflecting minimal potential interference. Serum samples displayed the largest variability (average S.D. = 4.88%) due to protein and lipid adsorption altering interfacial kinetics; nevertheless, recoveries remained acceptable (93.07–108.05%). Environmental water showed intermediate precision (average S.D. = 4.48%), likely from humic acids and suspended particulates.

In Figure 5E, the correlation between the proposed transient profiling method and the gold-standard ion chromatography (IC) technique was further evaluated using thirty samples with Ca^2+^ concentrations randomly distributed between 10 μM and 2 mM. In Figure 5E, linear regression yielded R^2^ = 0.997, indicating high agreement. The proximity of the slope to unity and the negligible intercept demonstrated that the single-exponential coefficient extracted from the 60 s transient window accurately reflected the true ion concentration without systematic bias. It is particularly noteworthy that such high correlation was achieved without requiring the prolonged conditioning (>1 h) typically mandated for conventional solid-contact ISEs. This finding underscored a key advantage of the present approach: by leveraging the intrinsic relationship between the transient potential decay rate and ion activity, the method bypasses the time-consuming equilibration process while preserving analytical accuracy comparable to laboratory-grade instrumentation.

This validation studies demonstrated that the transient potential profiling strategy not only achieves rapid detection (<1 min) but also satisfied the stringent reproducibility and accuracy requirements for practical ion sensing. Furthermore, we also compared the differences in response time and single detection time between our method and other existing electrochemical techniques in Table 1, highlighting the advantages of our method in rapid detection. The method’s performance across intra-day, inter-day, and inter-batch evaluations, combined with its successful validation against IC in real water samples, established a promising technological foundation for the development of conditioning-free, disposable ion-selective test strips for point-of-care and on-site environmental monitoring applications.

## 4. Conclusions

This work presented a rapid ion-quantification method that obviates the need for prolonged electrode conditioning by analyzing the transient potential decay within the first 60 s of polarization. A reverse-polarization step on indicator electrode, coupled with an optimized pre-polarization protocol, ensured a reproducible initial state, enabling the extraction of a concentration-correlated transient relaxation constant. The approach achieved excellent selectivity, pH tolerance, and reproducibility, with validation against the standard method showing high agreement across real samples. This strategy offered a practical paradigm for conditioning-free, disposable ion-selective sensors suitable for point-of-care and on-site environmental monitoring.

## Figures and Tables

**Table 1:** Transient potential profiling method comparison with other methods.

Method	Response Time	Single Detection Time	References
Transient Potential Profiling	5 s−10 s	<1 min	This method
Steady-state Potential Profiling	<40 s	10–30 min	[37]
Cyclic Voltammetry	<20 s	15–45 min	[38]
Chronoamperometry/Chronopotentiometry	<5 min	<15 min	[39]
Electrochemical Impedance Spectroscopy	<5 min	3–30 min	[40]

## Data Availability

The data presented in this study are available upon request from the corresponding author.

## Supplementary Materials

The following supporting information can be downloaded at https://www.mdpi.com/article/10.3390/bios16060335/s1: Table S1: Raw data from IC measurements; Supplementary Note S1: Definitions of repeatability and reproducibility tests. Figure S1. Characterization of the ion-selective electrode. (A) Water layer test (B) Selectivity co-efficients toward different interfering ions.

## Abbreviations

The following abbreviations are used in this manuscript:

ISEs	Ion-selective electrodes
RSD	Relative standard deviation
ETH 1001	Calcium ionophore IV
KTClPB	Potassium tetrakis(4-chlorophenyl)borate
o-NPOE	2-nitrophenyl octyl ether
PP	Polypropylene
PU	Polyurethane
PVC	Poly(vinyl chloride)
THF	Tetrahydrofuran
CaCl_2_·2H_2_O	Calcium chloride dihydrate
NaCl	Sodium chloride
KCl	Potassium chloride
MgCl_2_·6H_2_O	Magnesium chloride hexahydrate
DI	Deionized
SPCEs	Screen-printed carbon electrodes
SEM	Scanning electron microscopy
OCP	Open-circuit potential
IC	Ion chromatography
