# Supplementary material for: Transient Potential Profiling for Rapid Calcium Ion Quantification: Eliminating Conditioning Time in Solid-Contact Ion-Selective Electrodes
Source: Biosensors (Basel). 2026 Jun 12;16(6):335. doi: 10.3390/bios16060335 (PMC13297531; doi:10.3390/bios16060335)
Supplement: Supplementary file 1 [file biosensors-16-00335-s001.zip › biosensors-4315879-supplementary.pdf]

Supporting Information

Transient Potential Profiling for Rapid Calcium Ion Quantification: Eliminating Conditioning Time in Solid-Contact Ion-Selective Electrodes-Supporting Information

Kaijie Zheng 2, Chenjie Yan 2, Mengwei Jiang 2, Jing Lei 1, Chengcheng Wang 1, Kai Zhao 1,Dajing Chen 2,*Min Guo 2,*

Table S1. Raw data from IC measurements.

Sample No.	ICP-MS (mM)	Peak Area (a.u.)
1	0.012	-0.128
2	0.013	-0.125
3	0.013	-0.12
4	0.016	-0.115
5	0.019	-0.105
6	0.022	-0.096
7	0.029	-0.075
8	0.032	-0.064
9	0.034	-0.057
10	0.035	-0.055
11	0.048	-0.013
12	0.050	-0.007
13	0.069	0.053
14	0.082	0.096
15	0.089	0.119
16	0.106	0.172
17	0.121	0.220
18	0.127	0.239
19	0.193	0.449
20	0.196	0.461
21	0.302	0.797
22	0.372	1.023
23	0.396	1.100
24	0.634	1.860
25	1.141	3.480
26	1.253	3.836
27	1.333	4.091
28	1.678	5.195
29	1.767	5.480
30	1.872	5.816

Supplementary Note S1

Definitions of repeatability and reproducibility tests:

(A) Intra-day repeatability: multiple measurements on the same sensor within one day.

(B) Inter-day repeatability: measurements on the same sensor on three different days.

(C) Inter-batch reproducibility: measurements using sensors prepared from three independently fabricated batches.

(D) and (E) refer to (D) sensor to sensor variation within the same batch and (E) operator to operator variation.

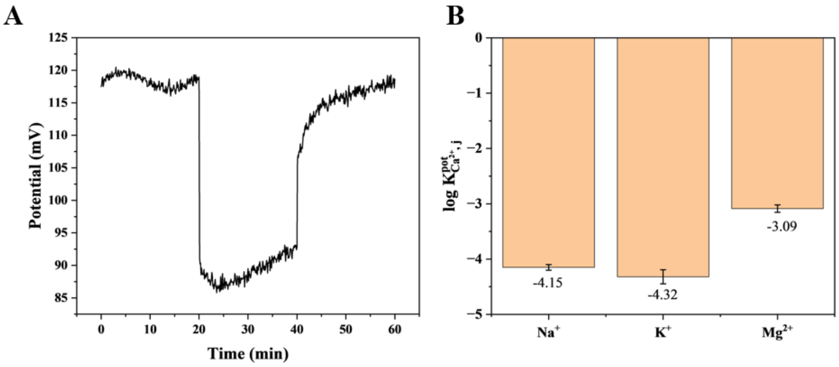

Figure S1. Characterization of the ion-selective electrode. (A) Water layer test (B) Selectivity coefficients toward different interfering ions.
